# A preinstalled nasopharyngeal airway in the right nasal passageway to facilitate nasogastric intubation in anesthetized and intubated patients: a prospective randomized controlled trial

**DOI:** 10.1186/s12876-020-01514-6

**Published:** 2020-11-03

**Authors:** Ting-Yang Huang, Jr-Rung Lin, Yung-Tai Chung

**Affiliations:** 1 Department of Anesthesiology, Chang Gung Memorial Hospital, Linkou Medical Center, No. 5, Fuxing St., Guishan District, Taoyuan, 33305 Taiwan; 2 Clinical Informatics and Medical Statistics Research Center and Graduate Institute of Clinical Medicine, Chang Gung University, Taoyuan, Taiwan

**Keywords:** Nasopharyngeal airway, General anesthesia, Endotracheal intubation, Nasogastric intubation, Deep sedations, Intensive care units

## Abstract

**Background:**

Nasogastric intubation (NGI) is usually challenging in patients under general anesthesia, with reported success rate at the first attempt to be less than 50%. The aim of this study was to investigate whether a preinstalled nasopharyngeal airway (NPA) in the right nasal passageway can facilitate NGI in anesthetized and intubated patients.

**Methods:**

A prospective randomized controlled trial including 108 patients scheduled for elective intra-abdominal surgeries requiring a nasogastric tube (NGT) was conducted. Fifty-three patients were randomized to receive NGI through a preinstalled NPA in the right nasal passageway (Group NPA) and 55 patients to receive NGI via the right nostril (Group O). The primary outcomes were success rates of NGI at first attempt, success rates of NGI in accumulative attempts, durations of successful NGI at the first attempt and success rates of NGI for the rescuing methods. The secondary outcomes were bleeding incidence and hemodynamic changes induced by NGI.

**Results:**

Success rate of NGI at the first attempt was 83.0% in Group NPA compared with 47.3% in Group O [*P* < 0.001; absolute risk reduction (ARR) = 35.7%, 95% confidence interval (CI) = 19.1–52.4%; relative risk reduction (RRR) = 67.8%] and success rate of NGI in accumulative attempts (two attempts maximum) was 88.7% in Group NPA compared with 63.6% in Group O (*P* = 0.002; ARR = 25.0%, 95% CI = 9.7–40.4%; RRR = 68.9%). Duration for NGI successful at the first attempt in Group NPA was significantly longer than that in Group O (56.3 vs. 27.1 s; *P* < 0.001; Mean difference = 29.2 s, 95% CI = 20.0–38.4 s). Neither bleeding incidence nor hemodynamic response is significantly different between the two study groups.

**Conclusions:**

The study indicates a preinstalled NPA in the right nasal passageway facilitates NGI in anesthetized and intubated patients as an initial NGI method and as a rescuing method for patients undergoing two unsuccessful initial attempts of NGI without a preinstalled NPA. However, the NPA method proved to take more time than the routine method for NGI successful at the first attempt.

*Trial registration*: The study was conducted after receiving approval from Institutional Review Board of Chang Gung Memorial Hospital, Linkou branch, Taiwan (registration number 201800138A3; April 11, 2018), and the clinicaltrials.gov (NCT03697642; Principal Investigator: Ting-Yang Huang; Date of registration: October 4, 2018; https://www.clinicaltrials.gov/NCT03697642).

## Background

Nasogastric intubation (NGI) is commonly performed in patients for emptying stomach and preventing aspiration pneumonia. However, NGI in anesthetized and intubated patients is usually challenging, with reported success rate at the first attempt to be less than 50% for a routine method [1, 2]. A nasogastric tube (NGT) is made of polyurethane or silicone and will become more flexible when warmed up by the patient’s airway so that it tends to coil or kink while facing anatomic block during insertion [3, 4]. It has been reported that the most common sites of contact are piriform sinuses (46%) and arytenoids cartilage (25%) [4]. Hence, plenty of techniques have been carried out to improve NGI [2, 5–13], but they are not without complications. An auxiliary nasal/pharyngeal instrument may induce a higher rate of epistaxis or oral mucosal bleeding [2, 6]; forward traction of the larynx may occasionally cause vasovagal reflex due to compression of bilateral carotid arteries [14].

In the pilot study, 80 patients were divided into four groups (with or without a preinstalled nasopharygeal airway (NPA) through either of nasal passageways). We found the success rate of NGI at first attempt with a preinstalled NPA in the right nasal passageway to be 30% higher than that without a preinstalled NPA (85% vs. 55%). However, there was no such advantage for NGI with a preinstalled NPA in left nasal where success rate of NGI at the first attempt was 65%. The results imply that a preinstalled NPA in the right nasal passageway facilitates NGI. To prove this hypothesis, we designed a prospective randomized controlled trial to investigate whether a preinstalled NPA in the right nasal passageway can effectively facilitate NGI in anesthetized and intubated patients. In addition, we also intended to test the effectiveness of the method as a rescuing means for patients undergoing failed routine NGIs.

## Methods

This prospective randomized controlled trial was conducted in accordance with the Declaration of Helsinki after receiving approvals from Institutional Review Board of Chang Gung Memorial Hospital, Linkou Branch, Taiwan (registration number 201800138A3; April 11, 2018) and the www.clinicaltrials.gov (NCT03697642; Principal Investigator: Ting-Yang Huang; Date of registration: October 4, 2018; https://www.clinicaltrials.gov/NCT03697642). A written informed consent was obtained from each patient who enrolled in our study.

Patients with American Society of Anesthesiologists’ (ASA) physical status of I-III, aged between 20 and 85 years scheduled for elective intra-abdominal surgeries requiring an NGT for perioperative care enrolled in the study, except those with conditions like coagulopathy, history of skull base fracture, nose diseases, any esophageal or gastric pathologies, hemodynamic instability.

The enrolled patients were randomized to Group NPA (NGI with a preinstalled NPA in the right nasal passageway) or Group O (NGI via the right nostril without a preinstalled NPA) based on permuted block randomization. The computer-generated randomization sequence was created, using SAS® 9.4 (SAS Institute Inc., Cary, NC, USA) statistical software, with 1:1 allocation of random block sizes of 4. Each of the randomized patients was coded and each code was concealed in an envelope. Then, all the envelopes were kept by the statistician without clinical involvement in the study. After the patient was anesthetized, an envelope with a designated number was disclosed. All the NGI were executed by an anesthesiologist (Huang), who has performed hundreds of NGIs, and all the results were verified and recorded by a nurse anesthetist.

For all study participants, general anesthesia was induced with fentanyl 1 μg/kg, lidocaine 1 mg/kg, propofol 2 mg/kg and cisatracurium 0.2 mg/kg. Endotracheal intubation was executed 3 min after the induction. The endotracheal tube (ETT) was fixed at right mouth corner and the cuff pressure was kept at 20 mmHg after the position of the tube was confirmed by auscultation and capnography. Then, anesthesia was maintained at 1 MAC of end-tidal sevoflurane for at least 15 min before NGI.

The size of NPA (“Covidien” Mallinckrodt™) was selected in accordance with the nose-to-ear lobe distance (ID 7.0 for 127 mm, ID 7.5 for140mm, ID 8.0 for 152 mm, ID 8.5 for 159 mm) [15] and a 14F, 105-cm lubricated polyurethane NGT (“Symphon” Comforsoft) was used for every patient in the study. In addition, the proper length of the NGT (the nose-tragus-xiphisternum distance) was measured before each NGI. In Group NPA, when NGI was completed, the NPA would be withdrawn, cut longitudinally and freed from the NGT. According to the study design, NGI for each case in either group would be tried twice maximally, and the NGT would be cleaned and soaked in cold water for 30 s between attempts if necessary. A successful NGI was confirmed by aspiration of gastric contents or auscultation over epigastrium. For the patient undergoing two unsuccessful initial attempts of NGI, we were to try the method designate for the other study group, two attempts maximally, to rescue the NGI. A Macintosh laryngoscope with a pair of Magill forceps was used to rescue NGI if four attempts all failed.

The primary purpose of this study is to examine success rates of NGI at first attempt, success rates of NGI in accumulative attempts, durations of successful NGI at the first attempt and success rates of NGI for the rescuing methods. Duration of NGI was defined as the time taken from inserting an NGT through the right nostril to the predetermined distance. The secondary purpose is to examine bleeding incidence and hemodynamic changes induced by NGI.

### Statistical analysis

The sample size was calculated using the G-power 3.1.9.2 software. The results of the pilot study (20 cases in each of 4 groups) indicate that compared with the routine method NGI with a preinstalled NPA in the right nasal passageway can reach an approximate 30% improvement of success rate (from 55 to 85%) at the first attempt. Consequently, based on α (type I error probability) at the significance level of 0.05, at least 53 patients for each group should enroll in the study to achieve 90% power and to reject the null hypothesis as well.

Data from all patients were analyzed according to their assigned group by intention-to-treat principle. Continuous variables, presented as mean (standard deviation (SD)) and 95% confidence interval (CI) of mean difference were examined by Student *t* test; categorical and proportional data were examined by Person χ^2^ test or Fisher exact. All the aforementioned data were analyzed using SPSS v.24 (SPSS Inc., Chicago, IL, USA) and 95% confidence interval (CI) of the difference in proportions was computed by Microsoft Excel spreadsheet according to the method presented by Altman. A value of *P* < 0.05 was considered statistically significant.

## Results

**Fig. 1:**
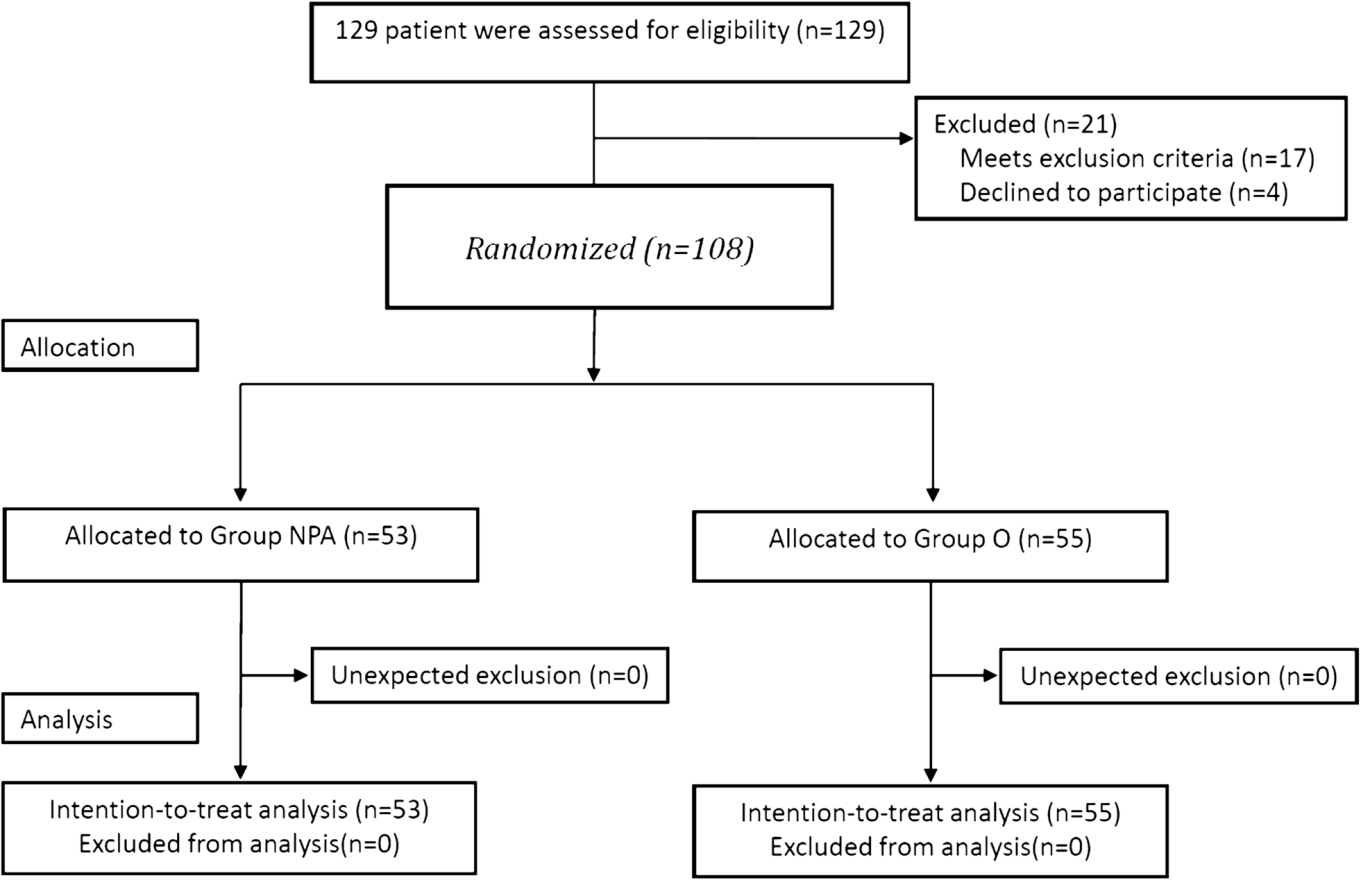
CONSORT diagram demonstrates patient recruitment and the flow of the participants. *NGT* nasogastric tube

One hundred and twenty-nine patients were assessed for eligibility from October 24, 2018 to September 3, 2019. Patients’ recruitment and the flow of the participants in the study are summarized in Fig. 1. One hundred and eight patients were eventually randomized to two groups: 53 patients for NGI with an NPA in the right nasal passageway (Group NPA) or 55 patients for NGI through the right nostril (Group O).

There is no significant difference between the demographic data of the two groups (Table 1). Success rate of NGI at the first attempt was 83.0% in Group NPA compared with 47.3% in Group O [*P* < 0.001; absolute risk reduction (ARR) = 35.7%, 95% confidence interval (CI) = 19.1–52.4%; Relative risk reduction (RRR) = 67.8%] and success rate of NGI in accumulative attempts (two attempts maximum) was 88.7% in Group NPA compared with 63.6% in Group O (*P* = 0.002; ARR = 25.0%, 95% CI = 9.7–40.4%; RRR = 68.9%) (Table 2). Duration of NGI successful at the first attempt in Group NPA was significantly longer than that in Group O (56.3 vs. 27.1s; *P* < 0.001; mean difference = 29.2 s, 95% CI = 20.0–38.4 s) (Table 2). For the cases undergoing two unsuccessful initial attempts of NGI, the method used in Group NPA reached a higher rescue rate than the method used in Group O (75.0% vs. 16.7%; *P* = 0.018; ARR = 58.3%, 95% CI = 23.0–93.7%; RRR = 70.0%) (Table 2). In addition, five patients in either group respectively required Macintosh laryngoscope and Magill forceps to complete NGI. The study outcome proved no significant difference in the total failure rates after 4 attempts in both groups (9.4% vs. 9.1%; *P* > 0.99; ARR = − 0.3%, 95% CI = − 11.3 to 10.6%) (Table 2). Neither bleeding incidence nor hemodynamic response is significantly different between the two study groups (Table 3). No patient in either group experienced any unexpected or severe complications perioperatively.

**Table 1:** Demographics of the study patients

Characteristics	Group NPA^a^ (*n* = 53)	Group O^b^ (*n* = 55)
Gender (male/female), n (%)	36/17 (68/32)	32/23 (58/42)
ASA I/II/III, n (%)	17/24/12 (32/45/23)	18/28/9 (33/51/16)
Age (years), mean(SD)	58.7 (13.8)	55.3 (13.8)
Height (cm), mean(SD)	163 (7.6)	162 (8.2)
Weight (kg), mean(SD)	66 (11.7)	62.3 (12.6)
BMI (kg/m^2^), mean(SD)	24.7 (3.4)	23.5 (3.8)
Tragus–nose tip*–*xiphisternum distance (cm), mean(SD)	56.2 (3.8)	55.6 (3.6)
Nose tip–earlobe distance (cm), mean(SD)	14.2 (1.1)	13.9 (1)
Neck circumference (cm), mean(SD)	36.2 (4.3)	34.9 (3.7)
Mallampati score I/ II/ III-IV, n (%)	23/25/5 (43.4/47.2/9.4)	30/17/8 (54.5/31/15.5)
Cormack–Lehane classification I/ II/ III-IV, n (%)	18/20/15 (34/38/28)	18/23/14 (33/42/25)

*ASA* American Society of Anesthesiologists’ Physical Status Classification, *NGI* nasogastric intubation, *BMI* body mass index, *SD* standard deviation

^a^NGI with an NPA in the right nasal passageway

^b^NGI through the right nostril

**Table 2:** Comparisons of success rates, rescue rates and durations of NGI successful at the first attempt

Outcomes	Group NPA^a^ (*n* = 53)	Group O^b^ (*n* = 55)	Statistical test results
Success rate at the first attempt, % (n/N)	83.0 (44/53)	47.3 (26/55)	*P* < 0.001 (Pearson χ^2^ test) ARR = 35.7, 95% CI = 19.1 to 52.4 RRR = 67.8, 95% CI = 38.6 to 83.1
Success rate in accumulative attempts (two attempts maximum), % (n/N)	88.7 (47/53)	63.6 (35/55)	*P* = 0.002 (Pearson χ^2^ test) ARR = 25.0, 95% CI = 9.7 to 40.4 RRR = 68.9, 95% CI = 28.6 to 86.4
Rescue rate for the failed cases by the method in the other group, % (n/N)	16.7 (1/6)	75.0 (15/20)	*P* = 0.018 (Fisher exact test) ARR = 58.3, 95% CI = 23.0 to 93.7 RRR = 70.0, 95% CI = 30.6 to 87.0
Total failure rate after 4 attempts, % (n/N)	9.4 (5/53)	9.1 (5/55)	*P* > 0.99 (Fisher exact test) ARR = − 0.3, 95% CI = − 11.3 to 10.6 RRR = − 3.8, 95% CI = − 238.0 to 68.1
Durations of successful NGI at the first attempt (s), mean (SD)	56.3 (32.5)	27.1 (8.6)	*P* < 0.001 (Student *t* test) Mean difference = 29.2, 95% CI = 20.0 to 38.4

*ARR* absolute risk reduction, *RRR* relative risk reduction, *CI* confidence interval, *NGI* nasogastric intubation

^a^NGI with an NPA in the right nasal passageway

^b^NGI through the right nostril

**Table 3:** Comparison of bleeding incidence and hemodynamic changes induced by NGI

Outcomes	Group NPA^a^ (*n* = 53)	Group O^b^ (*n* = 55)	Statistical test results
Bleeding, % (n/N)	5.7 (3/53)	12.7 (7/55)	*P* = 0.321 (Fisher exact test) ARR = 7.1, 95% CI = − 3.7 to 17.9 RRR = 55.5, 95% CI = − 63.0 to 87.9
ΔHR (BPM), mean (SD)	7.4 (9.6)	7.7 (11.3)	*P* = 0.884 (Student *t* test) Mean difference = − 0.3, 95% CI = − 4.3 to 3.7
ΔMBP (mmHg), mean (SD)	12.6 (12)	12.0 (12.9)	*P* = 0.813 (Student *t* test) Mean difference = 0.6, 95% CI = − 4.2 to 5.3

*ARR* absolute risk reduction, *RRR* relative risk reduction, *CI* confidence interval, *SD* standard deviation, *NGI* nasogastric intubation, Δ amount of change, *BPM* beats per minute

^a^NGI with an NPA in the right nasal passageway

^b^NGI through the right nostril

## Discussion

A routine NGI in anesthetized, paralyzed and intubated patients has been reported to reach a success rate to be less than 50% at the first attempt [1, 2] and unsuccessful attempts of NGI tend to increase complication rates [9, 10, 16]. Thus, various techniques have been designed to facilitate NGI. Maneuvers to stiffen an NGT for NGI include injecting water [9] into an NGT, freezing [10] an NGT, placing a “Rusch” intubation stylet [6] into an NGT and inserting an ureteral [2] or an esophageal [5] guidewire into an NGT. Other techniques such as neck flexion with lateral pressure [2], inflation of esophagus with air via a facepiece [11], forward traction of larynx [7, 8], utilization of GlideScope [12], slitting of a tracheal tube [2] and using a laryngoscope with Magill forceps [13] have also been reported. The aforementioned NGI methods bear success rates at first attempt to be between 66 and 99% [2, 5–8, 12], yet they also cause varied complications. Bleeding time can be as high as 22% for the slit tracheal tube method [2. Vasovagal reflex [14], bending of the endotracheal tube [6], regurgitation or even aspiration [11] is also likely to occur during NGI. Nonetheless, some of those NGIs require rather expensive and less accessible devices, an esophageal guidewire, for instance [17].

In our study, compared with Group O, NGI in Group NPA bore a significantly higher success rates both at the first attempt (83.0% vs. 47.3%) and in accumulative attempts (88.7% vs. 63.6%). The NPA method was also capable of rescuing NGI with a success rate to be 75% for cases undergoing two unsuccessful initial attempts of NGI that was performed without a preinstalled NPA. In the pilot study, an NPA in the right nasal passageway seems to be able to facilitate NGI, but the sample size is not enough to verify the effectiveness. Therefore, in this prospective controlled study, we enrolled sufficient cases to prove the advantage of an NPA in the right nasal passageway over the routine method with respect to NGI. In addition, we intended to know how the NPA method works as a rescuing method for cases undergoing failed routine NGIs. The longer time for NGI successful at the first attempt in Group NPA is likely to be related to friction generated between the NGT and the NPA during the insertion of the tube.

**Fig. 2:**
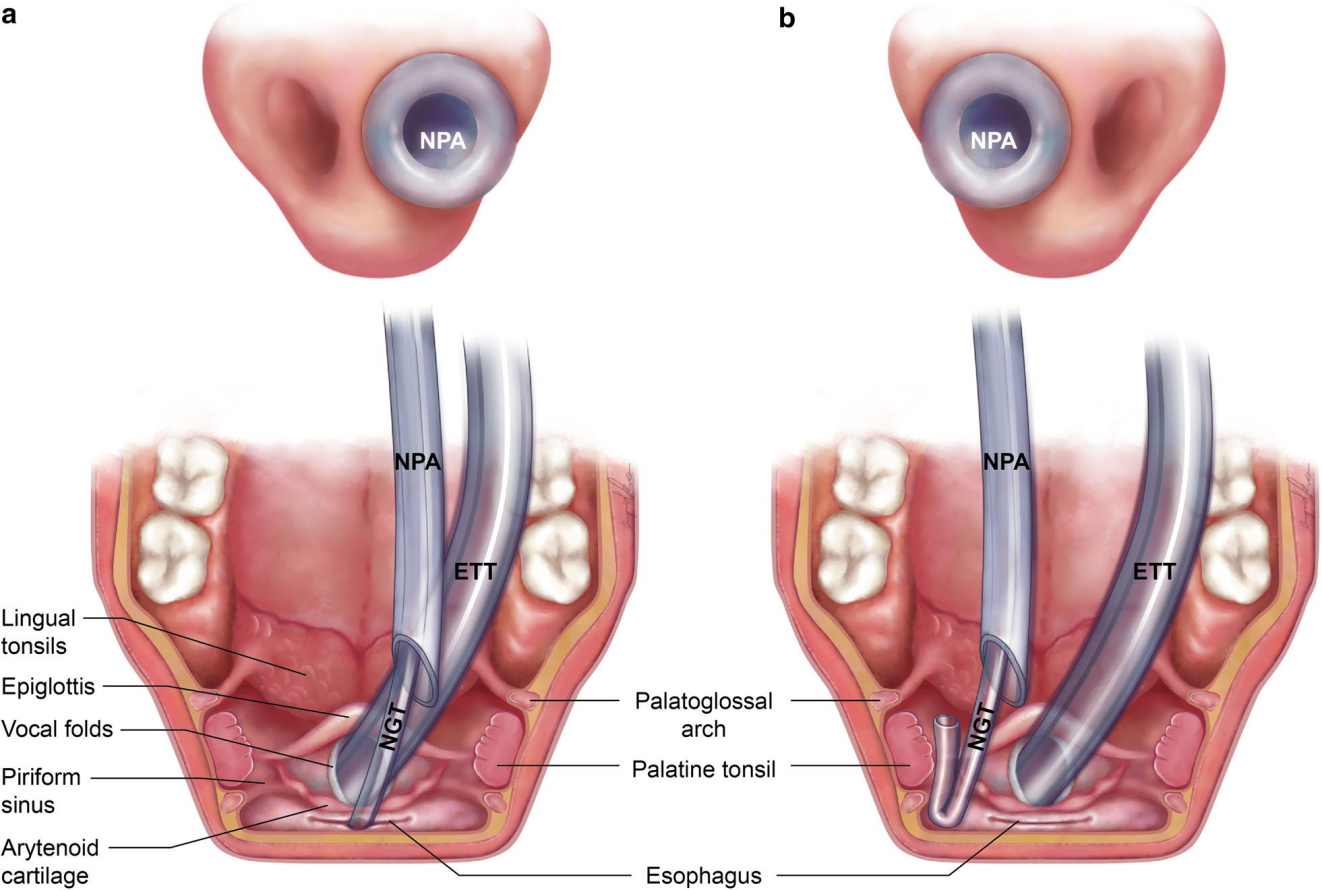
An NPA **a** in the right nasal passageway and **b** in the left nasal passageway as a conduit for an NGT during NGI (fully authorized to use the picture by the owner, Ingrid Kuo). *NPA* nasopharygeal airway, *NGT* nasogastric tube, *ETT* endotracheal tube, *NGI* nasogastric intubation

When an NPA is properly positioned, its distal end is usually past the tongue base and closer to the opening of esophagus. Moreover, with the bevel of the distal end of the NPA facing leftward, we noticed that, in vitro, the tip of the NGT has a propensity to detour leftward when it emerges from the distal end of the NPA. Therefore, an NPA in the right nasal passageway is able to offer an NGT a better chance to be advanced into the esophagus, which is illustrated in Fig. 2. The results of our study seem to echo this theory.

The method used in Group NPA is a readily learned technique and no neck manipulation or special devices are required. It can be applied not only to patients under general anesthesia, but also to paralyzed and intubated patients in intensive care units. Because of the NPA is soft and flexible, the entire procedure of NGI in Group NPA is minimally invasive and no unexpected or serious complications occurred periopeatively.

The results of this study are also subject to some limitations. First, the study only focused on patients with no pathologies from their nostrils to stomachs. Second, all the NPA insertions and NGIs were accomplished by the same physician, so personal biases could be unavoidable.

## Conclusions

In conclusion, a preinstalled NPA in the right nasal passageway facilitates NGI in anesthetized and intubated patients with a higher success rate at the first attempt, a higher success rate in accumulative attempts and a higher rescue rate. However, NGI successful at the first attempt with a preinstalled NPA takes more time than that without a preinstalled NPA. Furthermore, no significant difference has been detected between the two groups with regards to bleeding incidence and hemodynamic changes induced by NGI.

## Data Availability

The datasets used and analyzed during the current study are available from the correspondence author on reasonable request.

## Abbreviations

NGI: Nasogastric intubation
NPA: Nasopharygeal airway
NGT: Nasogastric tube
ETT: Endotracheal tube
ASA: American Society of Anesthesiologists’ Physical Status Classification
ARR: Absolute risk reduction
RRR: Relative risk reduction
CI: Confidence interval
SD: Standard deviation
Δ: Amount of change
BPM: Beats per minute
BMI: Body mass index
